# Characteristics of the Phytoplankton Community Structure and Water Quality Evaluation in Autumn in the Huaihe River (China)

**DOI:** 10.3390/ijerph182212092

**Published:** 2021-11-18

**Authors:** Yun Zhang, Wanli Gao, Yuying Li, Yeqing Jiang, Xiaonuo Chen, Yinlei Yao, Beata Messyasz, Kun Yin, Wenxiang He, Yong Chen

**Affiliations:** 1 International Joint Laboratory of Watershed Ecological Security and Collaborative Innovation Center of Water Security for Water Source Region of Middle Route Project of South-North Water Diversion in Henan Province, College of Water Resource and Environment Engineering, Nanyang Normal University, Nanyang 473061, China; zy174812@163.com (Y.Z.); jyq61359@163.com (Y.J.); chenxn1996@126.com (X.C.); yyl19892021@163.com (Y.Y.); messyasz@amu.edu.pl (B.M.); yinkun@cnemc.cn (K.Y.); gemc@gz.gov.cn (W.H.); cheny_110@163.com (Y.C.); 2 Department of Hydrobiology, Faculty of Biology, Adam Mickiewicz University in Poznan, Uniwersytetu Poznanskiego 6, 61-614 Poznań, Poland; 3 China National Environmental Monitoring Centre, Beijing 100012, China; 4 Guangzhou Environmental Monitoring Center, Guangzhou 510006, China; 5 Sichuan Environmental Monitoring Center, Chengdu 610091, China

**Keywords:** phytoplankton, diversity index, water quality, Huaihe River Basin

## Abstract

As an important indicator of phytoplankton in water quality evaluation, the phytoplankton community structure is very sensitive to changes in water quality, and analyzing their community composition and function is of great significance for the ecological management and maintenance of watershed environments. To understand the environment and ecological status as well as reconstruct or restore a healthy aquatic ecosystem in the Huaihe River Basin in China, a comprehensive phytoplankton survey was conducted in the main stream and main tributaries of the Huaihe River in 2019. A total of 266 species or genera of phytoplankton were identified, mainly belonging to Bacillariophyta and Chlorophyta. The number of phytoplankton species upstream and downstream was higher than that in the middle. The results of phytoplankton biomass showed significant spatial differences in different river reaches (*p* < 0.05). The identified phytoplankton functional groups (FGs) were divided into 27 groups, including 16 representative functional groups (RFGs), followed by A, B, F, G, H1, J, K, L_M_, L_O_, M, MP, P, T, T_B_, W_O_ and X2. The mean values of the Shannon–Wiener index and Margalef index were 2.47 and 2.50, respectively, showing that most of the water in the Huaihe River Basin was in a state of moderate nutritional status. The results of this study provided a reference for studying the composition and distribution of phytoplankton communities, nutrient status, and pollution levels in the Huaihe River Basin, as well as in other similar watersheds.

## 1. Introduction

Healthy water is one of the most important foundations for the sustainable development of human societies and ecosystems. With rapid economic development and population growth, water quality deterioration has become an important global problem, which may lead to the destruction of biodiversity, eutrophication and serious public health hazards. Spatiotemporal variation and trends in water quality can reflect geographical differences, sources of pollution and types of human activities [1]. Previous studies have reported that the water quality in different water bodies was easily affected by a wide range of factors, including climate change [2], natural sources [3] and anthropogenic activities, particularly mining [4], sewage discharge [5] and agricultural and urban industrial pollutants [6], while some synergistic effects can cause great changes in water quality [7], leading to insufficient dissolved oxygen and eutrophication, etc. The threat of various factors to the aquatic environment poses new challenges to the protection and management of water quality and quantity.

Phytoplankton are the main primary producers and one of the basic biological components of water ecosystems [8,9], and they have a wide distribution range and a short life cycle and play a crucial role in the energy flow, material cycle and information transmission in freshwater ecosystems [10,11]. The composition, population number and diversity of phytoplankton are important indicators of water quality, regarded as important indicators of river health [12]. Previously, phytoplankton have been used as a biological indicator to judge changes in water bodies, responding quickly to environmental changes [13]. By analyzing the characteristics of the phytoplankton community, the dynamic characteristics of water environmental quality can be comprehensively and timely grasped [12,14], which cannot be replaced by physical and chemical monitoring. At present, biological evaluation indexes and methods of water quality have been gradually introduced into river health evaluation systems and have become important for evaluating water pollution and nutrition levels. Some studies have investigated the use of phytoplankton for water quality assessment and other aspects [15,16,17,18]. Therefore, water quality assessments using aquatic biological are essential for understanding the impact of human activities on water quality and the heterogeneity of spatio-temporal characteristics, as well as for carrying out effective ecological protection.

As one of the seven major rivers in China, the Huaihe River is located at 111°55′–121°25′ E and 30°55′–36°36′ N and covers an area of 270,000 km^2^. This area is located in the transitional climate zone between warm temperate and subtropical zones and is characterized by a monsoon climate with distinct seasons [19]. The special geographical location and vast basin area cause the water quality of the Huaihe River to change both spatially and temporally. Due to the dense population and unbalanced industrial structure in the basin, the limited water capacity is seriously overloaded so as to deteriorate dramatically [20]. Ecological and environmental problems have become increasingly prominent, such as, for example, in the Huaihe River water pollution incidents of 1994 and 2004. Most of the studies on phytoplankton community structure and water quality evaluation in the basin have focused on tributaries, such as the Honghe River [21] and Yinghe River [22]. There have been few studies at basin scale that have evaluated water quality in the Huaihe River Basin via phytoplankton. Therefore, it is of great significance to understand the spatio-temporal variation in water quality and the main sources of influence to provide more effective water quality assessment methods for the environmental governance of water and public health. To analyze the distribution of the phytoplankton community and evaluate the trophic status of the Huaihe River Basin, as well as the current water quality situation, a systematic investigation and functional evaluation of phytoplankton in the Huaihe River Basin was conducted in October 2019. This study provides important theoretical support for water quality assessment, water ecosystem restoration and watershed sustainable management in the Huaihe River Basin and other similar basins.

## 2. Materials and Methods

### 2.1. Study Area and Sampling

**Figure 1:**
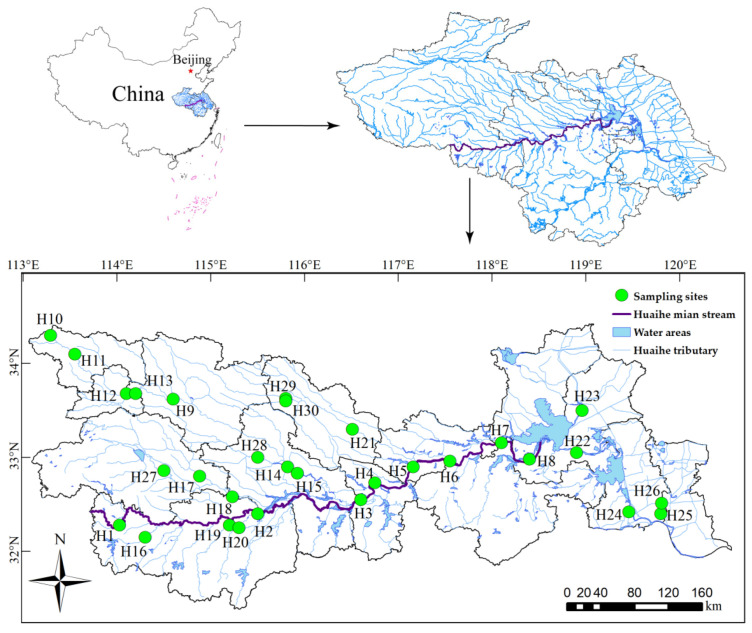
Distribution of sampling sites in the Huaihe River Basin.

Due to the obvious changes in the basin environment, the composition and distribution of the biological community in the basin also change significantly with the changes in river level. According to the distribution of the national automatic monitoring stations for surface water quality, 30 sampling sites were selected in the main stream and tributaries of the Huaihe River (Figure 1). Each river had at least one sampling site, following the main stream of the Huaihe River (H1–H8), Yinghe River (H10–H15), Shaying River (H9), Honghe River (H17–H18), Ruhe River (H27), Shihe River (H16), Huanghe River (H19), Bailuhe River (H20), Wohe River (H21), Quanhe River (H28), Youhe River (H30), Zhaowanghe River (H29), Yinjiang River (H25), Sanhe River (H22), Erhe River (H23), Beijing-Hangzhou Grand Canal (H24) and New Tongyang Canal (H26). H22–H24 and H26 sites belonged to natural rivers, and others belonged to artificial rivers. Among all sampling points, the west of H2 is the upper reaches of the Huaihe River, the east of H2 and the west of H22 are the middle reaches of the Huaihe River, and the east of H22 is the lower reaches of the Huaihe River. In addition, in this study, H1–H8 are the main stream of the Huaihe River, H9–H26 are the primary tributaries of the Huaihe River and H27–H30 are the secondary tributaries of the Huaihe River. 

### 2.2. Sample Collection and Determination Method

For qualitative phytoplankton collection, we collected phytoplankton samples by dragging a No.25 plankton net at a depth of 0.5 m for 3 min. The water samples concentrated in the mesh head were collected in a 100 mL sample bottle and fixed on site with 4% formaldehyde solution for microscopic inspection and identification [23].

For quantitative collection of phytoplankton, surface water samples were collected in a 2.5 L brown bottle at a depth of 0.5 m, and 15 mL of Lugol’s solution was immediately added for fixation. After bringing the samples back to the laboratory and allowing them to sit for 48 h, the water was concentrated to 40 mL using the siphon method, and microscope slides were used for the identification of phytoplankton to the genera or species levels (varieties).

For phytoplankton identification, the concentrated water samples were added to glass slides and placed under a 10 × 40 magnification microscope for observation, identification and photography. The qualitative samples were classified according to the morphological classification method, and species identification was conducted according to the records of freshwater algae in China [24].

Before algae identification and counting, the concentrated samples were shaken and transferred to a 0.1 mL counting board. Phytoplankton cells were counted under an inverted microscope BX53 (400×, Olympus Corporation, Tokyo, Japan) and converted to a per liter estimate. Each sample was counted twice. If the two counts differed by >15%, the cells were recounted again. Phytoplankton biomass was calculated with reference to the “Research Methods of Freshwater Plankton” [25].

### 2.3. Biological Water Quality Evaluation

The ecological assessment of the Huaihe River Basin was carried out by referring to the Shannon–Wiener diversity index (*H*′), Margalef diversity index (*d*) and the biodiversity index of the Huaihe River Basin (Table 1) [26].

The specific calculation is as follows:

(1) $$H^{\prime }=-\sum_{i=1}^{S}P_{i}\ln P_{i}$$

(2) d=(s−1)/lnN

In the above formulas, *P_i_* stands for the proportion of individuals belonging to the type of *i* in all individuals, *S* is the number of species and *N* is the total number of observed individuals.

### 2.4. Analytical Methods

Nonmetric multidimensional scaling (NMDS) can simplify the research objects in multidimensional space to low-dimensional space for positioning, analysis and classification while retaining the original relationship between objects. The NMDS graph usually gives the stress value of the model to determine whether the graph can accurately indicate the true distribution of the sorted data. Additionally, it is usually thought that stress <0.2 can be represented by a two-dimensional point diagram of NMDS, and its graph has certain explanatory significance; when stress <0.1, the sorting is good; when stress <0.05, the representation is good [27]. Kriging interpolation, also known as spatial local interpolation, is a method of unbiased optimal estimation of regional variables in a limited area based on variogram theory and structural analysis. In this study, the kriging interpolation method was used in ArcMap 10.7 software to analyze the spatial distribution and changes in phytoplankton. All statistical analyses and graphical processing of the data were carried out using Excel 2019 (Microsoft, Redmond, USA), SPSS 25 (IBM, Chicago, USA), Arc Map 10.07 (ESRI Redlands, USA Co., Ltd (ESRI, Redlands, CA, USA)) and R 4.0.4 software (The R Programming Language, The University of Auckland, New Zealand).

## 3. Results

### 3.1. Characteristics of the Phytoplankton Community Structure in the Huaihe River

**Figure 2:**
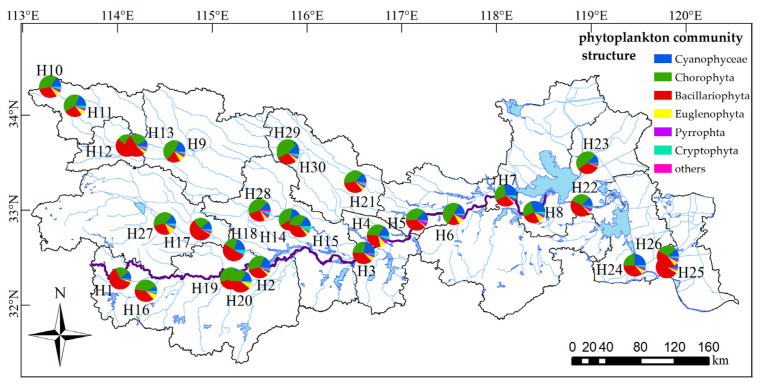
Composition of phytoplankton species in the Huaihe River Basin.

**Figure 3:**
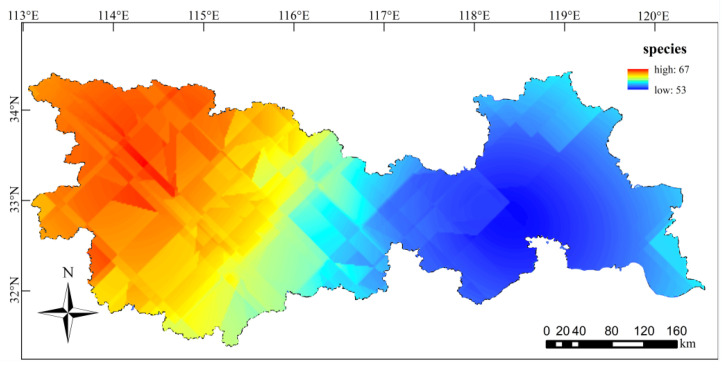
Variation in phytoplankton species in the Huaihe River Basin.

A total of 266 species of phytoplankton were found in 30 sampling sites in the Huaihe River Basin (Figure 2) belonging to 98 genera, 46 families and 8 phyla. Among them, 89 species of Bacillariophyta and 88 species of Chlorophyta accounted for 66.54%. There were 48 species of Cyanophyta (18.05%); the other 41 species of algae accounted for 15.41%. The spatial distribution of species number was significantly different (Figure 3, *p* < 0.05). The H11 sampling site had the largest number of species, i.e., 94, while the H8 sampling site had the smallest number of species, i.e., 23. The number of phytoplankton species in the upper and lower reaches was greater than that in the middle reaches, and the number of phytoplankton species in the upper reaches was much greater than that in the lower reaches.

**Figure 4:**
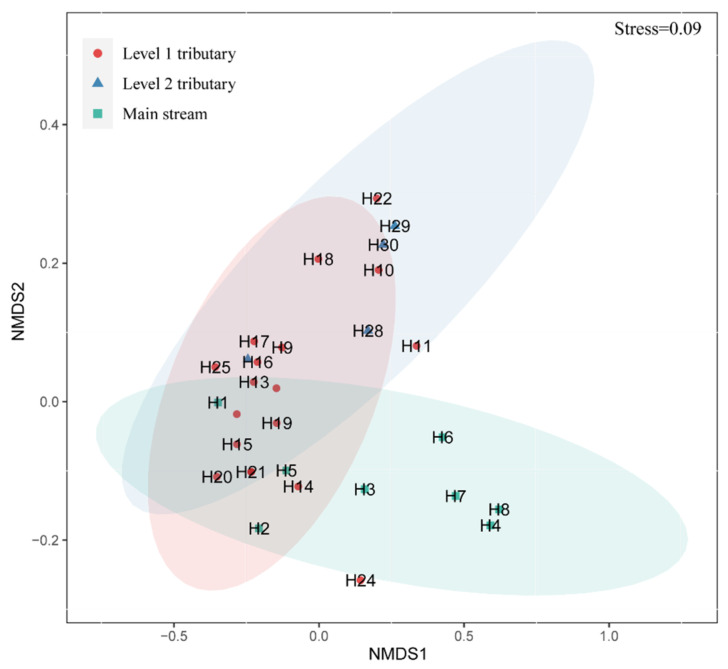
NMDS analysis based on phytoplankton density in the Huaihe River Basin.

NMDS can describe community differences between samples, so the closer the distance between the two samples, the more similar the species composition. NMDS analysis showed that there were significant spatial differences in phytoplankton community composition among the 30 sampling sites (Figure 4, *p* < 0.05). The difference was significant between the main stream and other tributaries of the Huaihe River, but there was no significant aggregation among the sampling sites in the main stream. The phytoplankton community composition of H1, H2 and H5 in the upper reaches of the main stream was more similar to that of the first tributary but less similar to that of other sampling sites in the main stream. The community composition of each sampling point of the primary tributary is significantly different (*p* < 0.05), and the community composition of H10, H11, H18 and H22 is similar to that of the secondary tributary.

### 3.2. Dominant Species of Phytoplankton in the Huaihe River

Species with dominances greater than 0.02 were selected as dominant species. The results showed that in October 2019, 34 dominant species of six phyla were found, mainly Chlorophyta and Diatoms (Table 2). *Cyclotella meneghiniana* sp., *Navicula* sp., *Synedra* sp. and *Cryptomonas erosa* appeared at higher frequencies. The dominant species in the Huaihe River were mainly β-mesotrophic, α-β-mesotrophic and α-eutrophic indicator species. There was a large difference in the number of dominant species in various river segments. The Ruhe River (H27) and Yinghe River upper stream (H11) had the most (nine) dominant species, among which the Ruhe River had *Ulnaria ulna*, *Cyclotella meneghiniana*, *Navicula* sp., *Chlorella vulgaris*, *Crucigenia tetrapedia*, *Scenedesmus* sp., *Desmodesmus communis*, *Cryptomonas erosa* and *Cryptomonas ovata*, and the Yinghe River had *Ulnaria ulna*, *Cyclotella meneghiniana*, *Nitzschia* sp., *Ulnaria acus*, *Pseudanabaena* sp., *Microcystis aeruginosa*, *Aphanizomenon* sp., *Oscillatoria* sp. and *Chroococcus minutus*. The dominant species of H8 in the lower reaches of the mainstream were the least, with three species (*Nitzschia* sp., *Microcystis aeruginosa*, *Cryptomonas erosa*).

### 3.3. Phytoplankton Biomass in the Huaihe River Basin

**Figure 5:**
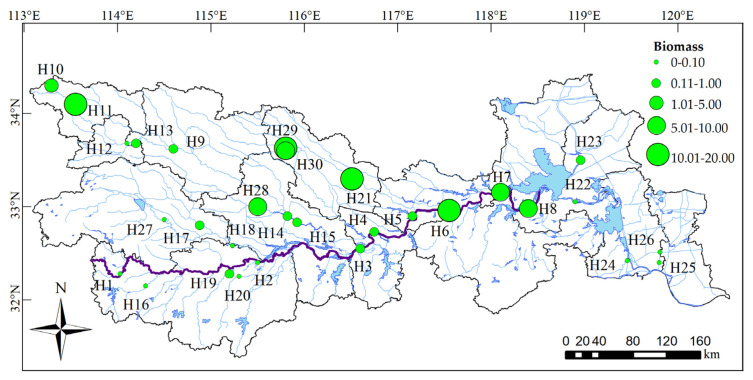
Phytoplankton biomass distribution in the Huaihe River Basin.

The maximum biomass of phytoplankton in the Huaihe River Basin was 18 mg/L, and the minimum was 0.01 mg/L (Figure 5). The average biomass was 3.20 mg/L. Chlorophyta, Cyanobacteria, diatoms and Cryptophyta contributed higher biomasses, which were 22.84, 17.89, 18.53 and 17.10 mg/L, respectively, accounting for 23.79%, 18.64%, 19.30% and 17.81% of the total biomass, respectively. Due to the different habitats, the biomass of phytoplankton at each sampling site also differed. Among the 30 sampling sites, the highest biomass occurred at H11 (18.00 mg/L), and the lowest biomass occurred at H2 (0.01 mg/L). Excessive biomass in some sampling sites should arouse the attention of management.

The results of the t test showed that the planar distribution of phytoplankton biomass was significantly different (*p* < 0.05). In terms of phytoplankton biomass, the middle and lower reaches of the main stream of the Huaihe River, the upper reaches of the Yinghe River and the reaches of the Wohe River, Youhe River, Zhaowanghe River and Quanhe River in Anhui Province had higher biomasses. The upper reaches of the main stream of the Huaihe River, the Shihe River, the Huang River, the Bailuhe River, the Ruhe River in the upper reaches of the Huaihe River, the Yinghe River in the lower reaches of the Huaihe River, the Beijing-Hangzhou Grand Canal and the Xintongyang Canal were low. Overall, the upper and lower reaches were lower, while the middle reaches were higher.

### 3.4. Phytoplankton FG in the Huaihe River

**Figure 6:**
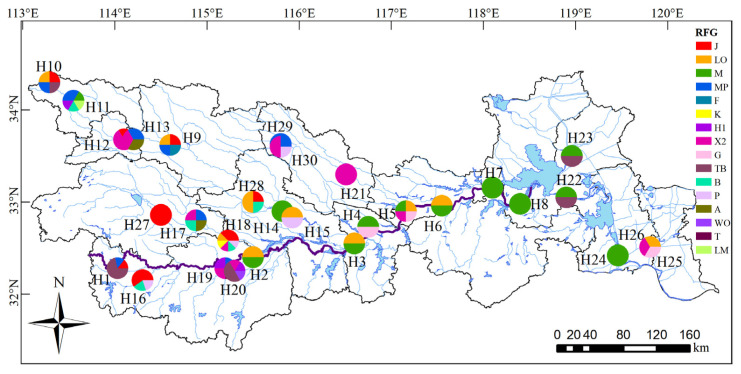
Distribution of phytoplankton functional groups in the Huaihe River Basin.

According to the phytoplankton functional group (FG) classification method proposed by Reynolds et al. [28] and Padisak et al. [29], these taxa were classified into 27 FGs, namely, A, B, C, D, E, F, G, H1, J, K, L_M_, L_O_, M, MP, N, P, S1, S2, SN, T, T_B_, Wo, W1, W2, X1, X2 and X3. According to the classification standard of the phytoplankton representative functional group (RFG) [30,31], the FG with a relative abundance greater than 5% was defined as the RFG of the sampling site. The RFGs were A, B, F, G, H1, J, K, L_M_, L_O_, M, MP, P, T, T_B_ (Figure 6), Wo and X2, and their distribution had obvious spatial differences. T_B_ was dominant in the upper reaches of the main stream, while M was dominant in the middle reaches, and the RFG was singular. In the tributary of the Yinghe River, MP was the main RFG, while in the Hongru River, J was the main RFG. In the southern mountainous area of the upper reaches, there were more J and T_B_ RFGs. In the middle reaches of the Huaihe River, X2 and L_O_ were the RFGs, and the RFG of the downstream artificial channel was M.

### 3.5. Biological Evaluation of Water Quality in the Huaihe River Basin

**Figure 7:**
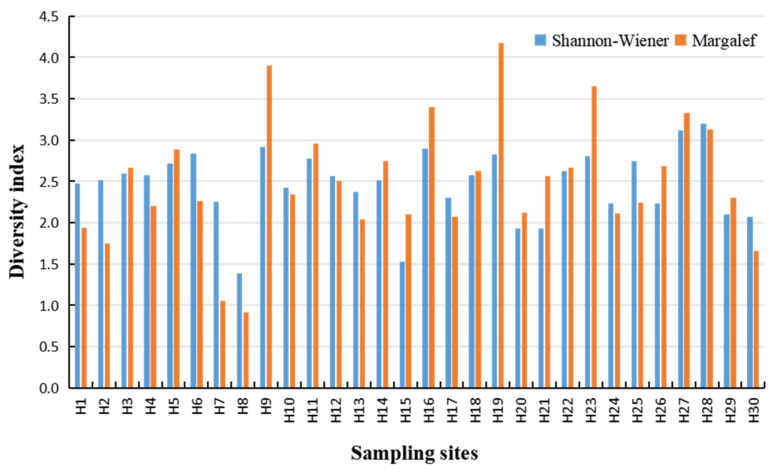
Phytoplankton biodiversity index in the Huaihe River Basin.

The Shannon–Wiener index of phytoplankton in the Huaihe River Basin fluctuated in the range of 1.39–3.20 (Figure 7), with an average of 2.47. According to the Shannon–Wiener diversity index, 28 sampling sites in the Huaihe River Basin had medium pollution, accounting for 93.3% of the total sampling sites, and two sampling sites had light or no pollution, accounting for 6.7% of the sampling sites. The Margalef diversity index ranged from 0.91 to 4.18, with an average of 2.50. According to the Margalef diversity index, one sampling site in the Huaihe River Basin had heavy pollution, accounting for 3.3% of the total sampling sites; 23 sampling sites had medium pollution, accounting for 76.7.3% of the sampling sites; and six sampling sites had light or no pollution, accounting for 20% of the total.

The Shannon–Wiener and Margalef indexes of the sampling sites were low in the lower reaches of the main stream of the Huaihe River. H8 was located between Xuyi County and Huaihe town. Extensive human settlement along rivers has caused substantial hydrological alterations that affect riverine community structure and function, and the Shannon–Wiener and Margalef indexes reached the minimum value. Using water quality biology to evaluate the water at this site, the Shannon–Wiener index result was medium pollution, and the Margalef richness index result was heavy pollution. The diversity index of sampling sites in the upper and middle reaches was higher, the highest value of the Shannon–Wiener diversity index appeared in H19, and the highest value of the Margalef richness index appeared in H28. In general, the phytoplankton community structure in the upper and middle reaches of the Huaihe River was complex and rich in species.

## 4. Discussion

### 4.1. Phytoplankton Community Structure and Biomass

A total of 266 species of phytoplankton were found in the Huaihe River Basin and main streams, of which Chlorophyta and Bacillariophyta accounted for 66.54%. Hong Song reviewed more than 100 papers, research reports, investigation reports and environmental quality reports concerning the aquatic communities in Chinese rivers since the 1950s, collected almost all existing biological monitoring data of major rivers in China [32] and concluded that diatoms were the dominant phytoplankton in Chinese rivers. Their result is different from our result; the reason for this difference may be related to the large amount of pollutant discharged into the Huaihe River in the past few decades, leading to the deterioration of water quality and serious damage to the aquatic ecosystem. The Huaihe River is located in the transition zone of a subthermal monsoon climate to a temperate monsoon climate, which is suitable for the growth of algae. Compared with 244 species of phytoplankton found in the spring of 2013 in the Huaihe River Basin [33], more species were identified at this time. This was the result of the local government’s promotion of industrial restructuring, intensified pollution prevention and control efforts and improved water quality in the river basin. Compared with rivers at other latitudes, the Huaihe River Basin had more species than that in the Lhasa River Basin and Yellow River with 53 species [34] and 114 species [8], respectively. However, the Huaihe River Basin had fewer species than that in the Haihe River Basin with 505 species [35]. Hong Song’s study had shown that diatoms were the dominant species of phytoplankton in Chinese rivers, among which *Melosira* sp. was the dominant species in most rivers [32]. Bacillariophyta and Chlorophyta were the dominant species in the Huaihe River Basin, which was different from the results of Hong Song’s study. It is generally believed that Dinophta, Cryptophyta and Bacillariophyta tend to dominate in mesotrophic water, while Chlorophyta and Cyanobacteria tend to dominate in eutrophic water [36]. The reason why the Huaihe River was dominated by Cyanophyta and Chlorophyta was that the Huaihe River is a typical densely populated river in China, and a large amount of domestic sewage and industrial wastewater flows into the Huaihe River, resulting in a high degree of eutrophication in the water. Zhu Weiju et al. obtained similar results with a study of phytoplankton in the Huaihe River Basin [37]. The frequency of phytoplankton, including *Cyclotella meneghiniana* and *Cryptomonas erosa*, the widespread distribution of which represents the eutrophication of water, indicated that most rivers in the basin were polluted to a certain extent. The distributions of *Cyclotella meneghiniana* and *Cryptomonas erosa* indicated that the eutrophication of water was widespread and that most rivers in the basin were polluted to a certain extent.

The distribution pattern of species reflects the amount of spatial resources occupied by species; on the other hand, the pattern indicates the distribution status of species in a habitat. It is generally believed that widely distributed species occupy many resource sites and have strong adaptability to diverse habitat resources. In the Huaihe River Basin, the species of *Cyclotella meneghiniana* and *Cryptomonas erosa* were extremely widely distributed, which indicated that these species had strong adaptability to the environment. With a large surface area and volume, *Cyclotella meneghiniana* could quickly absorb nutrients from water and grow. Wang Zhicong’s studies on the niche of dominant species of phytoplankton in Chaohu Lake showed that the niche width of *Cryptomonas erosa* was large, and this species had a strong ability to use resources and a wide range of ecological adaptations [38]. In the Huaihe River Basin, the distribution range of some phytoplankton-dominant species was narrow, such as *Mougeotia* sp. and *Coscinodiscus* sp.; they occupied a relatively concentrated resource point, and their biomass was higher in Anhui Province; this distribution was mainly due to these algae growing well in eutrophicated water, suggesting that the eutrophication degree of the middle reaches of the Huaihe River was more serious.

The phytoplankton biomass in the upper and lower reaches of the Huaihe River was lower than that in the middle reaches. More studies have shown that hydraulic conditions (such as flow or residence time) are the main factors influencing phytoplankton biomass and community composition [39]. The upstream channel had a faster flow rate, which made it more difficult for phytoplankton to attach to other substances in the water, leading to a lower biomass in the high-velocity channel [40]. The middle reaches accepted nutrients from the upper reaches, with a stable flow, slow flow rate and less suspended matter in the water, which were suitable for the growth of cyanobacteria [41]. In addition, most of the sampling sites in the middle reaches were distributed in populated areas such as cities and towns. Phytoplankton were affected by production and domestic water, showing the characteristics of higher biomass and a lower number of species, which was also a reflection of the high eutrophication degree of the water in the middle reaches. On the other hand, eutrophic rivers could form cyanobacterial blooms similar to lakes [42]. In seven catchments in the United Kingdom, the ecological risk associated with nuisance algal growth in rivers was largely linked to soluble reactive phosphorus concentrations during ecological sensitivity (low-flow periods) when biological activity was the highest [43]. In this regard, the phosphorus management strategy in the Tangxi River has been proven to be an effective way to control algal blooms [44]. The density of algae in some reaches of the Huaihe River has reached the level of severe algal blooms. Therefore, to prevent the continuous deterioration of water quality, it was necessary to strengthen the control of nutrients in the water, especially phosphorus. Most of the downstream reaches were artificial channels, which were greatly affected by human factors and had low water transparency, and light was the main factor driving the growth of phytoplankton [33].

### 4.2. Phytoplankton FG in the Huaihe River Basin

The division of phytoplankton FGs had a strong indication of the aquatic environment. A total of 27 FGs were identified, and 16 RFGs were identified in the Huaihe River Basin. Located in the upper reaches of the main stream and small tributaries in mountainous areas, the terrain had a large drop, and the river flowed quickly. RFG T_B_ was mainly adapted to the strong rapids of streams and rivers. The habitat type of *Microcystis* sp. represented by RFG M was stable eutrophic and super-eutrophic water with better transparency. Studies have shown that turbulence can prevent *Microcystis* sp. blooms and reduce depositional losses of nonbuoyant phytoplankton [37]. The closure and flow reduction resulting from many sluice gates and dams in the Huaihe River led to the weakening of the water exchange capacity, the accumulation of nutrients and an increase in water transparency. In addition, the inflow of urban sewage and industrial wastewater in the middle reaches has provided sufficient N, P and other nutrients for the growth and reproduction of *Microcystis* sp., which made *Microcystis* sp. form a good competitive advantage in a stable water environment. The abundance of *Microcystis* sp. was a manifestation of water eutrophication. The research results on the phytoplankton community structure in the Huaihe River Basin in spring [33] also proved this viewpoint. The MP RFG mostly appeared in shallow lakes with frequent disturbances and turbidity. The Yinghe River is located in Henan Province and has a dense population and numerous sluices and dams, and the river is greatly disturbed by human activities [45]. Dense dams have slowed the flow rate, resulting in a decline in the self-purification capacity of the river and the transformation of the phytoplankton community structure from river type to lake type, thus allowing the MP to become the RFG in this area. *Chlamydomonas* and *Cryptomonas* are representative species of RFG X2, and their habitat is medium to eutrophic shallow water. Dinophta were the representative species of RFG Lo and had wide adaptability. The sampling sites in the middle reaches were concentrated in the middle reaches of the Wohe River and the lower reaches of the Yinghe River. The flow was low, thereby accepting nutrients from the upper and middle reaches of the river, and the flow was suitable for the growth of Cryptomonas. Some flagellate groups (*Cryptomonas*, *Dinophta* and *Chlamydomonas*) rely on the swimming of flagella to find a small environment suitable for their own growth so that they can quickly absorb nutrients and grow [33], thereby occupying a dominant position. This indicates that the eutrophication degree of the water in this region was higher, and Cryptophyta and Dinophta contributed higher biomass, which also proved this result. The lower reaches of the Huaihe River were mostly artificial channels, which have been greatly disturbed by human activities. Abundant nutrients such as N and P provide favorable conditions for the growth and reproduction of *Microcystis* sp. Studies have shown that RFG M, X2 and J were mainly suitable for growing in eutrophic water [46]. Yi Qitao studied the driving factors of phytoplankton FG in shallow eutrophic lakes of the lowland areas of the Huaihe River, and the results showed that RFG X2 in Cryptophyta was the only dominant RFG that responded to the increased nutrient concentration (total nitrogen and total phosphorous), which became abundant mainly in the spring and autumn [47]. This indicated that RFG X2 rapidly grew with increased nutrients and thus dominated. The emergence of RFGs such as M, X2 and J indicates that eutrophication in the Huaihe River was more serious and that there was a greater ecological risk.

### 4.3. Phytoplankton Diversity and Water Quality Evaluation

Phytoplankton are the primary producers of aquatic ecosystems, and they can quickly respond to changes in the nutritional status of the water and the invasion of pollutants in the water [48]. Therefore, the phytoplankton diversity index is often used to evaluate the status of phytoplankton communities and water pollution [49,50]. It is generally believed that the higher the phytoplankton diversity index is, the better the water quality [51]. The phytoplankton diversity index showed that most of the sampling sites in the basin were in a state of medium pollution, except some sampling sites in the upper reaches of the river, and H8 was in a state of heavy pollution (Table 1), which was consistent with the research results of Qiu Yangling et al. [52]. Zhu Weiju et al. used a single aquatic biological index to evaluate the water quality in the Huaihe River Basin, and the results showed that the water was in a medium to heavy pollution state [33]. This result was different from the results of this study and the causes of such differences are as follows. Since 2006, the state and the local government of the Huaihe River Basin have increased the regulation of the Huaihe River. Combined with the “Water Special” water science and technology innovation and engineering management synergy, the government has accelerated the comprehensive treatment of the Huaihe River, and the Huaihe River water quality has improved. Zhao Kun et al. used the rotifer diversity index to evaluate the water quality in the Huaihe River Basin and found that most of the river reaches were heavily polluted [53]. Zuo Qiting et al. evaluated the health status of water bodies in the middle and upper reaches of the Huaihe River by using the water ecological health evaluation system and the comprehensive pollution index of water quality. The results showed that the water ecology in the middle and upper reaches of the Huaihe River was seriously degraded, and 60% of the monitored sections were at the “ill” or “sub-ill” level [54]. Zhang Ying et al. showed that most of the water bodies in the Huaihe River Basin were in an unhealthy state through the evaluation results of the integrity index of benthic animals [55]. This was related to the fact that the Huaihe River Basin is a large population-intensive basin in China, where domestic sewage and industrial wastewater were abundant and provided sufficient nutrients for the reproduction of phytoplankton. However, different river reaches are affected by different human activities, and different phytoplankton demands and utilization characteristics of nutrients are different [56], which is also one of the reasons for the difference in phytoplankton community structure and population density in the Huaihe River Basin.

## 5. Conclusions

A total of 266 species (including varieties) of 8 phyla, 98 genera and 46 families were identified in the Huaihe River Basin. They were mainly Chlorophyta and Bacillariophyta. However, the biomass was dominated by Chlorophyta, Bacillariophyta, Cyanophyta and Cryptophyta. Phytoplankton communities in the main stream of the Huaihe River differed greatly (*p* < 0.05). Phytoplankton biomass was also significantly different in different river reaches (*p* < 0.05), and the distribution of phytoplankton in the entire watershed showed significant spatial differences (*p* < 0.05). The phytoplankton index showed that the water quality of the studied reach was in a state of medium pollution.

The construction of the dam and sluice gate has changed the flow velocity, sediment content and nutrient distribution of the Huaihe River system, which has substantially changed the aquatic ecological conditions of the Huaihe River. The algae density in some sampling sites has reached the level of severe algal blooms, which should be considered by water ecological management. Although some achievements have been made in the treatment of the Huaihe River, the problem of water ecology is very serious, and the eutrophication of the water in the basin is still not optimistic. Aquatic ecosystems still need strengthened management and protection. Monitoring and controlling the content of nutrients in the water and improving the eutrophication of the water are the focus of current work.

This study was conducted only in autumn and did not cover all seasons. There may be errors in the survey results due to the influence of climate, water quantity and other changes. Given the layout of the sampling sites, the distribution density of the sampling sites was small, and the layout refers only to the water quality and hydrological stations, which may lead to the problem of substituting points for surfaces. In future work, the above problems will be improved.

## Figures and Tables

**Table 1:** Criteria for the ecological evaluation of water quality.

	Heavy Pollution	Medium Pollution	Light or No Pollution
*H*′	0–1	1–3	>3
*d*	0–1	1–3	>3

**Table 2:** Dominant species of phytoplankton in the Huaihe River Basin.

Category	Dominant Species	Frequency	Category	Dominant Species	Frequency
Bacillariophyta	*Ulnaria ulna*	19/30	Dinophta	*Gymnodinium* sp.	23/30
	*A. granulata* var. *angustissima*	1/2		*Peridinium* sp.	1/4
	*Cyclotella meneghiniana*	1	Euglenophyta	*Euglena* sp.	17/30
	*Aulacoseira granulata*	1/2	Chlorophyta	*Closterium* sp.	23/30
	*Navicula* sp.	8/9		*Chlamydomonas* sp.	3/5
	*Gomphonema* sp.	17/30		*Mougeotia* sp.	1/10
	*Cymbella* sp.	2/5		*Oocystis* sp.	11/15
	*Fragilaria* sp.	3/5		*Eudorina* sp.	8/15
	*Synedra* sp.	5/6		*Chlorella vulgaris*	11/30
	*Nitzschia* sp.	2/3		*Crucigenia tetrapedia*	1/2
	*Coscinodiscus* sp.	7/30		*Scenedesmus* sp.	2/3
	*Melosira varians*	4/15		*Desmodesmus communis*	2/3
	*Ulnaria acus*	4/15	Cyanophyta	*Chroococcus* sp.	17/30
	*Cocconeis placentula*	7/15		*Microcystis aeruginosa*	2/5
Cryptophyta	*Cryptomonas erosa*	23/30		*Pseudanabaena* sp.	17/30
	*Cryptomonas ovata*	7/10		*Aphanizomenon* sp.	2/5
	*Cryptomonas* sp.	7/30		*Oscillatoria* sp.	13/30

## Data Availability

Not applicable.
